# Clusters of Sociodemographic Characteristics and Their Association with Food Insecurity in Mexican University Students

**DOI:** 10.3390/foods13162507

**Published:** 2024-08-10

**Authors:** Pablo Alejandro Nava-Amante, Alejandra Betancourt-Núñez, Andrés Díaz-López, María Fernanda Bernal-Orozco, Ulises De la Cruz-Mosso, Fabiola Márquez-Sandoval, Barbara Vizmanos

**Affiliations:** 1 Doctorate in Translational Nutrition Sciences, Department of Human Reproduction, Growth and Child Development Clinics, Centro Universitario de Ciencias de la Salud (CUCS), Universidad de Guadalajara (UdeG), Guadalajara 44340, Jalisco, Mexico; pablo.nava@alumnos.udg.mx (P.A.N.-A.); fernanda.bernal@academicos.udg.mx (M.F.B.-O.); ulises.mosso@academicos.udg.mx (U.D.l.C.-M.); yolanda.marquez@academicos.udg.mx (F.M.-S.); bvizmanos@yahoo.com.mx (B.V.); 2 Institute of Nutrigenetics and Translational Nutrigenomics, Department of Molecular Biology and Genomics, Centro Universitario de Ciencias de la Salud (CUCS), Universidad de Guadalajara (UdeG), Guadalajara 44340, Jalisco, Mexico; 3 Laboratory of Nutritional Status Evaluation, Department of Human Reproduction, Growth and Child Development Clinics, Centro Universitario de Ciencias de la Salud (CUCS), Universidad de Guadalajara (UdeG), Guadalajara 44340, Jalisco, Mexico; 4 Doctorate in Public Health Sciences, Department of Public Health, Centro Universitario de Ciencias de la Salud (CUCS), Universidad de Guadalajara (UdeG), Guadalajara 44340, Jalisco, Mexico; 5 Center for Educational Research and University Welfare, Department of Philosophical, Methodological and Instrumental Disciplines, Centro Universitario de Ciencias de la Salud (CUCS), Universidad de Guadalajara (UdeG), Guadalajara 44340, Jalisco, Mexico; 6 Immunonutrition and Nutritional Genomics Network in Autoimmune Diseases, Department of Neurosciences, Centro Universitario de Ciencias de la Salud (CUCS), Universidad de Guadalajara (UdeG), Guadalajara 44340, Jalisco, Mexico; 7 Nutrition and Mental Health Research Group (NUTRISAM), Faculty of Medicine and Health Sciences, Universitat Rovira i Virgili (URV), 43201 Reus, Spain; andres.diaz@urv.cat

**Keywords:** food security, food insecurity, sociodemographic characteristics, university students, college students, cluster analysis

## Abstract

Food insecurity (FI) expresses irregular access to sufficient, safe, and nutritious food. We analyze the association between clusters of sociodemographic characteristics and FI in university students from Mexico. The Latin American and Caribbean Food Security Scale was applied, and household type, socioeconomic status (SES), and the characteristics of the head of the household, among others, were asked in a cross-sectional study. We performed Two-Step cluster analysis and logistic regression models. We included 466 university students (72.5% women, 47% with FI). We identified three clusters; Cluster-1 (*n* = 163) included a single-parent (37.4%) or co-resident/roommate (27%) household type, middle SES (63.2%); the head of the household is usually a woman (76.1%), single (71.8%), and with bachelor’s degree (41.7%) or less educational level (46.6%). Cluster-2 (*n* = 144) included the nuclear (89.6%) household type, high SES (76.4%); the head of the household is usually a man (79.2%), in a relationship (99.3%), and with a bachelor’s degree (63.2%) or graduate level (33.3%). Cluster-3 (*n* = 147) is very similar to Cluster-2. The difference is that Cluster-3 includes middle SES (70.1%), and the head of the household’s educational level is high school or less (100%). Only belonging to Cluster-1 was positively associated with FI (OR = 1.96, 95%CI: 1.24, 3.09). These results show the interrelationships between multiple sociodemographic characteristics and should contribute to developing interventions that simultaneously address numerous sociodemographic factors to diminish FI in university students.

## 1. Introduction

According to the Food and Agriculture Organization of the United Nations (FAO) food insecurity (FI) “is present in individuals when they lack regular access to sufficient, safe and nutritionally adequate food, the consumption of which allows for normal growth and development, with the goal of leading an active and healthy life” [1].

The COVID-19 pandemic favored the increase in FI prevalence. Compared to 2019, in 2022, the prevalence of moderate FI or severe FI increased from 25.3% to 29.6% globally, and from 31.5% to 37.5% in Latin America and the Caribbean [2]. In Mexico, these FI levels also increased, from 22.6% in 2018 to 25.9% in 2021 [3].

FI affects the university population. In college students from the United States (U.S.) [4,5,6], Australia, Canada, Malaysia, and South Africa [6], the average prevalence of any level of FI is around 32–42% (ranging from 9.9% to 84%). In Mexico, we analyzed the prevalence of FI in a representative sample of households with university students (data of 2018), and FI was 30.8% (95%CI: 29.7, 31.8%) [7]. FI in college students, however, may have increased due to the pandemic [8]. To our knowledge, no other studies analyze the prevalence of FI among university students in Mexico.

Experiencing FI in college has been positively associated with poor academic performance [9]; poor self-perceived health [10]; poor sleep quality [11]; obesity or overweight [12]; the use of substances such as alcohol, tobacco, vaping, and illicit drugs [13]; depression and anxiety [10]; stress [11]; emotional eating [14]; and being diagnosed with any eating behavior disorder [15]. In addition, FI was negatively associated with both dietary diversity [16] and adherence to the Mediterranean diet [17]. In Mexican university students, mild, moderate, or severe FI has been inversely associated with adherence to dietary patterns composed of healthy foods (fruits, vegetables, and animal protein [fish, dairy, and meat]) [18].

Studies carried out on college/university students from the US [4,6,10,19,20,21,22,23], Australia [6,24,25], South Africa [6], Canada [6], Turkey [26], Lebanon [27], Nigeria [28], and Mexico [7] have identified the sociodemographic predictors of FI such as belonging to a racial or ethnic minority [4,6,7,10,19], having children [24], being an undergraduate student [20,25], not receiving any type of scholarship [20], and being in the first years of study [7]. Age was positively [19] or negatively associated with FI [7,26]. Furthermore, having low economic income [26,27,28] or low socioeconomic status [7], receiving student loans [20], being financially independent, not receiving food assistance, or receiving financial aid in general [21] or from family [20], not being in the habit of keeping a budget [21], and being a worker [7,10] were all positively associated with FI. However, working to become financially independent has been inversely associated with FI in college students [19]. Concerning student housing, FI is more likely to occur when living in a shared household with roommates (off-campus) [22] or in a rented household [25], as well as whether the head of the household is female [7] or has a low level of education [7,23]. All the studies individually analyze the sociodemographic characteristics associated with FI. We have not identified a study that analyzes sociodemographic variables as a whole. 

Characterizing sociodemographic factor patterns in university students can support understanding interrelationships (i.e., co-occurrence and interaction) between multiple sociodemographic characteristics. Ultimately, this can contribute to developing guidelines and interventions that simultaneously address numerous sociodemographic factors in university students. Moreover, studies on sociodemographic characteristics applying exploratory, data-driven techniques, such as cluster analysis, are sparse [29], particularly among university students. Furthermore, we have only identified one study that analyzes the sociodemographic factors associated with FI in university students in Mexico, and this study explores the variables individually. One of the main differences between Mexican university and university students from other geographical contexts is that in Mexico, most of them live with their parents or relatives during their university education [7]. This is because universities are located in cities, and most students tend to access them without having to move, as mobility is a concept that is little practiced in the country in relation to university studies. This could make a difference in the results observed in the other studies. Studies on this issue in this context are needed to support future research with a higher level of scientific rigor.

Therefore, we aimed to analyze the association of clusters of sociodemographic characteristics and FI in Mexican university students.

## 2. Materials and Methods

### 2.1. Study Design and Participants

This cross-sectional study is part of a macro project that aims to analyze the FI risk factors and their consequences on lifestyle and cardiovascular health in workers and students at a public university in Mexico. In this macro project, we used non-probabilistic convenience sampling to recruit participants. The invitation to the study was made in person by study collaborators at a university campus in the healthcare area. To invite undergraduate students, we attended four groups per semester in each study area (nutrition, medicine, psychology, nursing, physical culture and sports, dentistry, and higher university technician). The selection of the groups was arbitrary; we only ensured that there were two groups from the morning shift and two from the afternoon shift. Approximately 3980 to 4975 students were invited to the study, as we visited 199 groups, each with 20–25 students. Graduate students in the health field and undergraduate students in non-health fields who heard about the study and wanted to participate were also included. Regarding workers, we attended workplaces where access was authorized. Those who voluntarily wanted to participate completed an online registration. As for the students, although we invited the same number of students in each degree program, students from some degree programs, like medicine and nutrition, participated more than others. Likewise, even though men and women received the invitation to the study equally, we had a greater involvement of women than men. 

Using online questionnaires sent to macro project volunteer participants (via email or WhatsApp^®^), we collected data on sociodemographic characteristics, FI, eating behavior, food consumption, sleep quality, and emotional state. Subsequently, we obtained anthropometric, clinical, and biochemical data in a face-to-face manner. The questionnaire’s content is described before displaying the questions. It is mentioned that participation in the study is voluntary and that their answers will be kept confidential. They were given a contact number to clarify any future doubts about their participation or about the research project. After these detailed specifications, a question was added in which the participants selected whether they agreed to answer the questions. If the participants agreed to participate, the questions were presented; otherwise, the questionnaire remained hidden, their participation ended, and their initial interest was acknowledged. Subsequently, in a face-to-face appointment, those who completed the online questions were invited to formalize and sign a letter of informed consent, documenting what they had already accepted online. All the participants recorded their responses using a numerical code per participant.

This study adheres to the Declaration of Helsinki and was approved by the Research, Research Ethics, and Biosafety Committees of the Centro Universitario de Ciencias de la Salud of Universidad de Guadalajara (approval number CI-02322).

For the present analysis, we included volunteers, regardless of their gender, aged 18 years or older, active university students from a public university in Mexico, for whom we had complete information on sociodemographic characteristics and the Latin American and Caribbean Food Security Scale (ELCSA, its acronym in Spanish). The data for the present study was collected from April 2022 to November 2023. 

### 2.2. Sociodemographic Characteristics 

Using online questionnaires with multiple-choice questions, we collected the following sociodemographic characteristics of university students: sex, age, marital status, school year, degree program, type of student (undergraduate or graduate), self-ascription to an Indigenous group, the receipt of some type of scholarship or food support, work activity, presence of children, and the person who supports them financially.

We also asked for the following characteristics of the university student’s household: essential services (potable water, gas, and electricity at home), homeowner, parents’ educational level, the number of heads of the household, as well as sex, marital status, and the academic level of the head of the household. If the household had two or more heads of the household, we described the information of the head of the household who registered first. We categorized the type of household according to who the student lives with, as unipersonal (the student reports living alone), co-resident (the student reports living with roommates who are not their relatives), nuclear (the student reports residing with both parents and with/without siblings), single parent (the student reports living with the father or mother and with/without siblings), or extended (the student reports living with other relatives, with/without parents/siblings). We also measured the socioeconomic status of the household with a six-question questionnaire with response options validated in the Mexican population. The Asociación Mexicana de Agencias de Inteligencia de Mercado y Opinión (AMAI-2020) prepared this questionnaire, which includes questions about 1. the educational level of the head of household; 2 to 4. the number of complete bathrooms, cars, and rooms used for sleeping; 5. members aged ≥14 years with labor activity; and 6. the presence or absence of internet at home. Based on the participants’ responses, the socioeconomic status was classified, in decreasing order, into A/B, C+, C, C−, D+, D−, and E [30].

Most of the sociodemographic variables included in this study were taken from the questionnaires of the National Survey of Household Income and Expenditure (ENIGH) [31]. ENIGH is a national Mexican survey that provides information on the sociodemographic characteristics of the Mexican population and the characteristics of their homes. These variables were also analyzed in a previous analysis of university students in Mexico [7]. Other variables, such as type of student, student’s financial support, students’ children, and the reception of food aid, were taken from studies that analyzed the association of sociodemographic variables with FI in university students [20,21,22,24,25]. All the sociodemographic characteristics were selected based on previous studies that analyze the factors associated with FI in university students from various geographical contexts, considering the limited evidence on university students in Mexico. These questions were initially sent to 16 participants (pilot testing) to evaluate their clarity. Once the clarity of the questions was confirmed, the study began, and the questionnaires were sent to the participants.

### 2.3. Food Security and Insecurity Status

We evaluated the state of food security or FI with the ELCSA. This instrument has been validated in the Mexican population [32] and consists of 15 dichotomous questions (yes = 1 point and no = 0 points). This questionnaire is aimed at finding out if, in the last three months, due to the lack of money or other resources, any member of the household presented certain situations, such as worrying about food running out, running out of food, skipping mealtimes, feeling hungry but did not eat, having a low variety food consumption, etc. [33]. The first eight questions of the ELCSA explore the food access situation of adults living in the household. The remaining seven questions probe the situation of children under 18 in the household (only answered if there are children). The levels of food security and FI in households without and with children under 18 are as follows: food security (0 points), mild FI (1–3 or 1–5 points, respectively), moderate FI (4–6 points or 6–10 points, respectively) and severe FI (7–8 or 11–15 points, respectively) [33]. For this analysis, we recategorized this variable into two categories: food security and FI (grouping mild FI, moderate FI, and severe FI). In this study sample, the ELCSA obtained a Cronbach’s alpha of 0.906 in the questions answered by those who only have adults in their household, and a Cronbach’s alpha of 0.869 in the questions responded to by those who have people under 18 years of age in their household. These values confirm the internal consistency of ELCSA in the study population, as in the general population [32,33].

### 2.4. Statistical Analysis

We evaluated the normality of the quantitative variable age with the Shapiro/Wilk test. The distribution of the variable was non-normal; therefore, we presented the variable as median and 25th and 75th percentile. The qualitative variables are presented as frequencies and percentages.

We analyzed the differences in food security and FI according to the sociodemographic characteristics. For this purpose, we used the Mann/Whitney U test to compare age according to FI. We also used Chi-square or Fisher’s exact test, as appropriate, to compare the qualitative variables.

We performed a Two-Step cluster analysis with the sociodemographic variables; we selected Log-likelihood as a distance measure and Schwartz Bayesian (BIC) as clustering criteria [34]. The variables that remained in the cluster analysis were selected based on (a) the literature, (b) the results obtained in the regression analyses, (c) the interpretation of the clusters, and (d) based on the results of the Two-Step cluster that showed the variables that best grouped or characterized the participants.

Subsequently, we analyzed the difference in the sociodemographic characteristics between the clusters with Chi-square. To explore the differences in the frequency of FI between the clusters, Chi-square was used. Then, the column proportions were compared with the z-test to identify which clusters the difference was found. The *p*-values of the multiple comparisons were corrected with the Bonferroni method.

To analyze the association between the sociodemographic characteristics, as individual factors and as clusters (independent variables), and FI (dependent variable) we performed simple and multiple logistic regression analyses. We present these results as Odds Ratio (OR) and 95% confidence intervals (95%CIs).

We included in the multiple logistic regression model those variables with a value of *p* < 0.2 in the simple logistic regression. We also assessed multicollinearity with the variance inflation factor (VIF). In this analysis, a VIF value > 2.5 suggests multicollinearity problems [35]; accordingly, we eliminated from the model those variables that, although they had a value of *p* < 0.2 in the simple logistic regression, had a VIF greater than this cutoff point.

In the case of the association analysis between the clusters and FI, we did not perform any adjustments.

We have performed the cluster analysis in SPSS v. 21 for Windows (IBM, Armonk, NY, USA) and the rest of the statistical analyses in STATA v. 15.1 (StataCorp, College Station, TX, USA). We consider a value of *p* < 0.05 as a significant result.

## 3. Results

### 3.1. Sociodemographic Characteristics

In Table 1, we present the participants’ personal sociodemographic characteristics; in Table 2, the characteristics of the students’ households can be seen. We included a total of 466 college students. The median age of the participants was 20 years, and 72.5% were female. Most students were undergraduates (93.3%) and were in their first two years of study (63.5%). The undergraduate majors with the highest frequency of participants were nutrition (22.8%) and medicine (20.5%). Almost a third part of the voluntary students worked (33.1%), one-sixth received some scholarship (15.2%), and many fewer had some kind of food support (3%), had children (2.6%) and self-ascription to an Indigenous group (0.4%). It was more common for the student’s parents or guardian to be one of the financial supporters (87.1%) (Table 1).

Regarding the household characteristics of the university students (Table 2), slightly more than half of them lived with both parents (nuclear household, 59.4%), or lived in a home owned by their family (61.7%), and were in the higher socioeconomic statuses (35.2% [level A/B], 34.6% [level C+]). Almost all the households had essential services such as electricity (99.8%), drinking water (98.5%), and gas (97.2%). Also, almost all the households had a refrigerator and stove for food storage and preparation (99.6%, respectively). However, in 3.4% of the households, there was at least one failure in essential services or household appliances. Of the heads of household, 54.5% reported having only one head of the household, 57.7% were male, 73% were in a relationship, and 50% had an educational level of high school, technical or less. Interestingly, only 12.9% of the fathers and 10.1% of the mothers had a doctorate/master’s degree (Table 2).

### 3.2. Frequency of Food Security or Food Insecurity by Sociodemographic Characteristics

**Figure 1:**
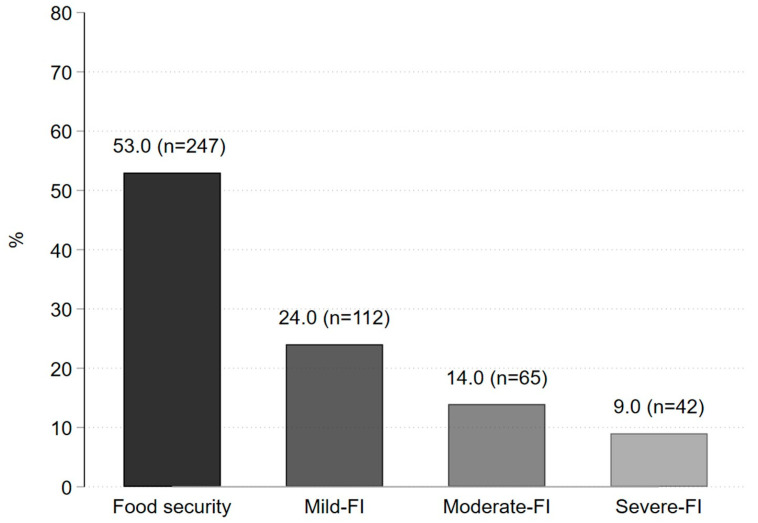
Frequency of food security/insecurity in university students according to the Latin American and Caribbean Food Security Scale (*n* = 466).

The FI frequency in university students was 47% (24% mild FI, 14% moderate FI, and 9% severe FI) (Figure 1). We identified significant differences in the FI frequency according to some student’s sociodemographic characteristics. A higher frequency of FI is observed in those students who work (55.2%), study psychology (65.6%) or some higher technical university degree (64.3%), live in a household as a co-resident or roommate (63.1%), do not or sometimes have some essential services or appliances (81.3%), in which there is only one head of household (52%) or in households in which the head of the household is woman (53.8%). Also, there is a tendency that for a higher frequency of FI, the educational level of the head of household, the father or the student’s mother, is lower (Table 1 and Table 2).

### 3.3. Association between Individual Sociodemographic Characteristics and Food Insecurity

The sociodemographic characteristics significantly associated with FI or that had a *p* < 0.2 in the simple regression analysis are shown in Table 3. The results of the simple logistic regression of all the sociodemographic variables (with or without a significant *p*-value) are shown in Table S1.

The sociodemographic variables that maintained a significant association with FI in the multiple logistic regression model are the following: the student working was associated with 1.55 times more probability of having FI (OR = 1.55, 95% CI: 1.03, 2.35). Regarding the student’s household, FI was 64% more likely when the head of the household was female (OR = 1.64, 95%CI: 1.07, 2.51), 95% more likely when the head of the household had a bachelor’s degree education (OR = 1.95, 95%CI: 1.04, 3.66), or 136% more likely if they had a high school/technical education or less (OR = 2.36, 95%CI: 1.28, 4.33). In contrast, living in an extended household was 65% protective of FI (OR = 0.35, 95%CI: 0.16, 0.76) (Table 3).

### 3.4. Clusters of Sociodemographic Characteristics of University Students

We identified three clusters based on the eleven sociodemographic variables analyzed. Nine of these variables had a *p* < 0.2 when analyzing their association with FI. We added the other two variables because they contribute to the differentiation of the clusters. The importance of each sociodemographic variable in the characterization or differentiation of clusters is presented in Figure S1. The sociodemographic characteristics of each cluster are detailed in Table 4.

The students grouped in Cluster 1 (*n* = 163) are characterized by living in a single-headed household (83.4%). The head of the household is usually single (71.8%), women (76.1%), and has a bachelor’s degree (41.7%) or less than high school education (46.6%). The home is owned (45.4%) or rented (43.6%); the household type is single-parent (37.4%) or co-resident/roommate (27%). The socioeconomic status is mainly C+ (39.3%) or C (23.9%), which could be classified as middle socioeconomic status. The educational level of the student’s mother and father is high school or less (49.1% and 49.1%, respectively) or bachelor’s degree (44.8% and 40.5%, respectively). In this cluster, 20.2% of the students do not receive financial support from their parents or guardians, and 33.1% are working (Table 4).

In Cluster 2 (*n* = 144), two-thirds of the students have two or more heads of household (66%). The head of the household is very often in a relationship (99.3%), frequently is a man (79.2%), and their educational level is undergraduate (63.2%) or graduate (33.3%), a similar percentage to that of the father and mother (90.3% and 69.4%, respectively). The household in which the student lives tends to be nuclear (89.6%), usually homeowners (77.1%), and with socioeconomic status in the highest category (A/B; 76.4%). Regarding the students, 11.1% do not receive financial support from their parents or guardians, and 27.1% are working (Table 4).

Cluster 3 (*n* = 147) is characterized by having two or more heads of household (60.5%), with the majority being male (76.9%). All the heads of the household (100%) are in a relationship and have a high school education or lower. The household is nuclear (87.8%), with socioeconomic status C+ (42.9%) or C (27.2%), which could be interpreted as middle socioeconomic status. The house is owned (65.3%), and the father’s (94.6%) or mother´s educational level is high school or lower (91.8%). In this cluster, only 6.1% of the students are not financially supported by their parents or guardians, and 38.1% are working (Table 4).

**Figure 2:**
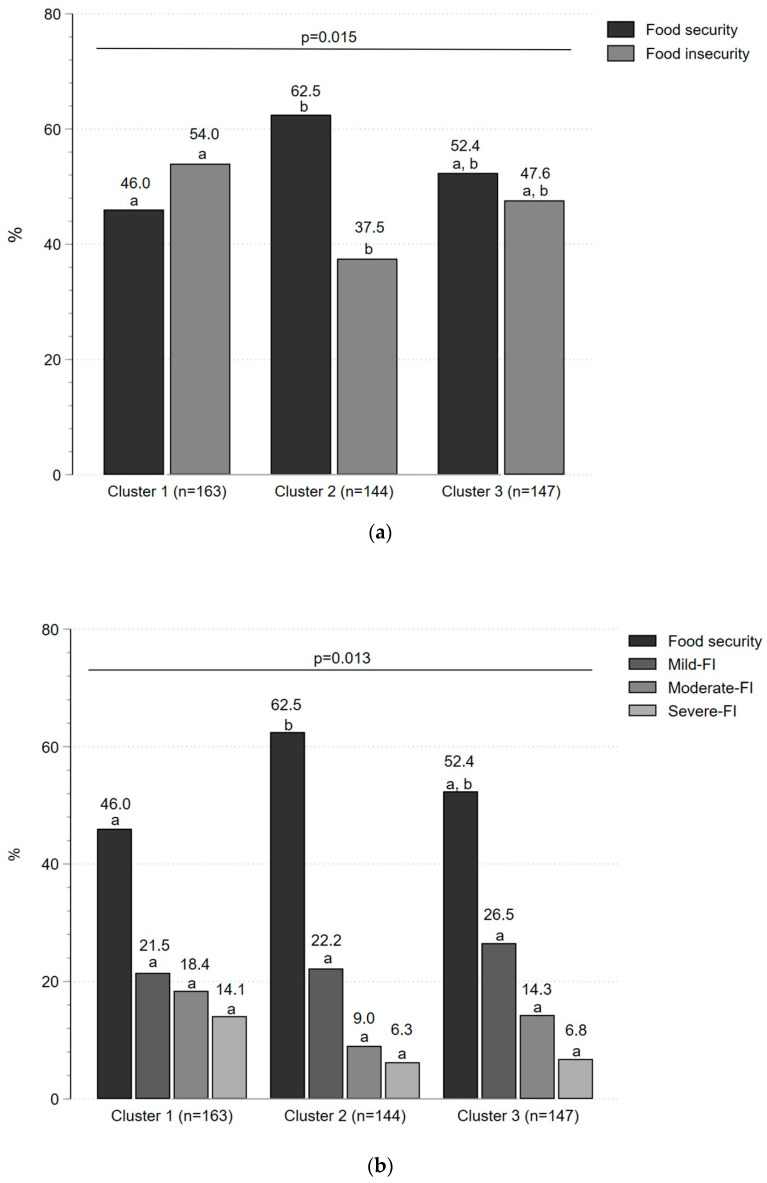
Frequency of food security/insecurity according to the three clusters of sociodemographic characteristics. (**a**) The frequency of food security/insecurity according to the clusters; (**b**) the frequency of food security and FI (mild, moderate, and severe) according to the three clusters. The difference between both the categorical variables was analyzed with Chi-square. We also compared the column proportions with the z-test. The values that do not share the same subscript letter differ significantly (*p* < 0.05).

When comparing the frequency of food security/insecurity among the clusters, we identified that FI was more common (54%) in Cluster 1 compared to Cluster 2 (37.5%) (*p* < 0.05) (Figure 2a). Furthermore, according to the degree of FI, there was no significant difference in the frequency of mild, moderate, and severe FI among the three clusters (Figure 2b).

### 3.5. Association between Clusters of Sociodemographic Characteristics and Food Insecurity

The students in Cluster 1 were 1.96 times more likely to present FI (OR = 1.96, 95%CI: 1.24, 3.09) with respect to those in Cluster 2. We observed a non-significant trend in the association between being in Cluster 3 and FI (OR = 1.52, 95%CI: 0.95, 2.42) (Table 5).

## 4. Discussion

In the present study, we identified three clusters based on some sociodemographic characteristics of university students, and we analyzed their association with the presence of FI. Cluster 1 was characterized by including, for the most part, students in whose household the head of the household is single, female, and with a bachelor’s degree or lower level of education. The type of household is single-parent or co-resident/roommate, and the socioeconomic status is mainly middle. Cluster 2 mostly grouped students in households where the head of the household is in a couple, is male, and his educational level is undergraduate or graduate; the household is nuclear and has a high socioeconomic status. Cluster 3 is similar to Cluster 2; the difference between the two is that in Cluster 3, the educational level of the head of household and the student’s parents is high school or lower, and the socioeconomic status is middle. Those who formed Cluster 1 were more likely to present FI taking Cluster 2 as a reference. No significant association was observed between belonging to Cluster 3 and presenting FI.

To our knowledge, this is the first study that analyzes the association of clusters based on sociodemographic characteristics with FI in any population group or context, in addition to analyzing individual characteristics. The richness of this analysis is that people have a set of sociodemographic characteristics that are very possibly associated with each other. That is, we do not have individual and isolated characteristics.

Thus, in the present study, we observed that belonging to Cluster 1 was positively associated with the presence of FI. This cluster comprises several characteristics that have been individually positively associated with FI. Previous studies conducted in U.S. households with minors [36,37], in the general Ethiopian [38], Zimbabwean [39], and in Mexican population [40] as well as in the U.S. [23], Germany [41], and Mexican [7] university students showed that the sociodemographic characteristics positively associated with FI are living in a single-parent household [36,37], that the head of the household is single [39,40], that the head of household is female [7,39,40], or that the parents [23,41] or the head of household [7,38,39] have a low educational level. Even in our study, the female head of the household or the head of the household with an academic level equal to or lower than a bachelor’s degree were also individual elements positively associated with FI.

One of the main explanations for why it is a risk factor that the household is headed by a woman, is single, and corresponds to a single-parent household is due to the wage gap between men and women. According to data from the United Nations, the global wage gap is around 20% and could increase for reasons that cause discrimination, such as skin color, being a migrant, or suffering from a disability [42]. Likewise, maternity can be a negative factor in women’s professional progression, affecting their economic income and, therefore, increasing the wage gap [42,43,44]. Particularly in Mexico, although women’s economic participation has increased in recent years [45], the wage gap with men is approximately 35% [46]. In this sense, when a woman heads a household, the economic income is usually lower, and, therefore, access to food can be negatively affected. Previous studies on Turkish [26], Lebanese [27], and American [28] university students have identified that low economic income is associated with FI.

Also, in Cluster 1, in most cases, the educational level of the household head and parents was a bachelor’s degree or lower. This is consistent with previous studies in the general population of Ethiopia [38] and Mexico [40] and also in Mexican university students [7], in which a low educational level of the parents or heads of the household is positively associated with FI. Previous reviews have described that when the academic level is higher, so is productivity and qualifications and, consequently, economic income [47,48]. In Mexico, recent data from the ENIGH-2022 indicate that economic income is directly proportional to schooling level [46]. The bachelor’s degree or higher education level represents a protection to present FI.

These arguments also explain why Cluster 2, whose households were characterized by having male heads of household, in a relationship and with a bachelor’s or graduate level of education, had a lower frequency of FI compared with Cluster 1. Cluster 2 was also characterized by nuclear-type households, and the students had a high socioeconomic status. These elements make it possible to maintain the household members’ quality of life and well-being [30] and probably protect them from FI.

Although it is true that in Cluster 2, most participants presented sociodemographic characteristics that were protective for FI, 37.5% is still a high frequency of FI if we consider that it is a relatively favored group. The presence of FI in this cluster could be because certain sociodemographic characteristics decrease the probability of presenting FI. However, the presence of these protective factors does not exempt people from not presenting FI. For instance, it could be that the head of household has a high level of education but does not practice what he or she has studied and, therefore, does not receive competitive salaries. Particularly in Mexico, it is common for some people with a bachelor’s degree not to find a job appropriate to their training, to work in positions that do not correspond to their training and academic qualifications, or to work in positions for which they are overqualified. Those who work in occupations that do not require a higher education degree or work informally are more likely to receive a lower salary than those with a job that matches their academic training. Also, it is the case that bachelor’s graduates, mainly women, decide not to participate in the labor market [49]. This same argument would explain why the FI risk factors observed in Cluster 1 did not result in 100% of the participants in this cluster having FI. In this case, undoubtedly, some women earn according to their academic training, without the distinction of sex. These results show the need to measure other sociodemographic variables, such as household income and the occupation of the head of household, which would allow us to have a broader picture of this problem.

On the other hand, Cluster 3 is similar to Cluster 2; the difference being that in Cluster 3, the household socioeconomic status is “middle” (not high, as in Cluster 2), and the educational level of the head of household and the students’ parents is high school or lower (not undergraduate or graduate as in Cluster 2). The presence of these two risk factors could explain why, in Cluster 3, the frequency of FI was slightly higher compared to Cluster 2, although the difference was not significant.

Other characteristics significantly associated with FI in this study, on an individual basis, were belonging to an extended household and the student’s work activity. In our analysis, we identified that university students who lived in extended households with other family members (with or without parents) were less likely to present FI. Like us, previous research on the general Mexican population has described that extended households offer protection for FI [40], and also, U.S. college students living with their parents or other relatives are less likely to have FI [50]. Extended households, having more members with possible economic activity, could have more financial income overall. This would prevent food access from being a problem.

Regarding work activity, this characteristic was positively associated with FI in this study. In agreement with our results, previous studies have reported that having a job in the university stage [7,10,23] represents more likelihood of FI. The reasons for working during college could be diverse; for example, some college students had part-time and full-time work as a coping strategy to obtain food [51]. Other important reasons why college students work include the need to help with family finances or obtain the income needed to continue studying [52]. In our study, one-third of the university students had work activity and presented a higher frequency of FI compared to those who did not work. The time allocated to work could compromise the time needed to take classes and schoolwork, a situation that could imply low academic performance [52,53].

In our study, most of the students’ personal sociodemographic characteristics, such as age, sex, year of study, etc., did not appear as protective or risk factors for FI. The reason household characteristics, and not individual characteristics, weighed more heavily in the association with FI could be due to the fact that most of the students in this study live in nuclear or extended households; that is, they live with their parents and/or relatives. This situation differs from that of other countries such as Australia and the US [22,25], where students leave home to live alone or with roommates and face other difficulties. The situation of not leaving home when studying at university could imply savings in maintenance and rent for students.

Finally, although it is true that our study sample is not representative of our study universe, it is relevant to highlight that almost half (47%) of the participants have some level of FI. This frequency is higher than that observed in previous reviews on FI in students from other geographic contexts (average FI between 32.2% and 42%) [4,5,6] and higher than the one reported in a national study of university students in Mexico (30.8% [95%CI: 29.7, 31.8%]) [7]. This higher frequency of FI may be due to the fact that our sample comes from a public university, a characteristic that has been positively associated with FI in other studies [7,27]. Also, our data collection occurred after the COVID-19 pandemic. Recent studies show that this pandemic increased the frequency of FI in university students [8,54].

One of the limitations of this study is the cross-sectional design, so causal relationships cannot be assumed. Another limitation is that the sample of this study is not representative of the study universe due to non-random sampling, so the results cannot be generalizable. Nevertheless, due to the limited evidence regarding the factors associated with FI in Mexican university students and the lack of evidence regarding the clusters of the sociodemographic characteristics related to FI, in addition to the usual analysis of individual variables, this article shows preliminary evidence that should be confirmed in longitudinal studies and with more extensive and random samples [55]. Many studies that analyze the factors associated with FI in university students from countries such as the US [4,10,19,20,21,22], Australia [24,25], Turkey [26], Lebanon [27], and Nigeria [28] are cross-sectional. However, some longitudinal studies can serve as examples for future studies with this study objective [23,56,57]. Likewise, the invitation to participate in the survey could be sent by electronic means (institutional social networks or institutional emails) as has already been carried out in previous studies [10,19,20,23,25,27], which favor that all the students have the same opportunity to participate. Furthermore, in future studies with a similar study objective, the presence of FI could be explored with more than one instrument to provide an opportunity to compare the results with other international studies. Many studies of college students measure FI using the USDA Household Food Security Survey Module [4,10,19,21,22,23,24,25,28,56,57], but there are other tools, such as the FAO Food Insecurity Experience [58]. Finally, considering the limited evidence that exists regarding the study of FI in university students from Mexico, it is suggested to conduct research regarding the consequences that FI has on academic performance, emotional state, diet, and health status, among others, as has already been performed in other contexts [10,23,24,26,27,56], in order to be able to prevent situations that can be improved with institutional policies based on accurate diagnoses.

## 5. Conclusions

Our findings suggest that if the household of Mexican university students, as a whole, is single-parent or co-resident/roommate, has a mainly middle socioeconomic status, and the head of the household is female, single, and with a bachelor´s degree or less education, there is a higher probability of presenting FI. This information is relevant to understanding this problem, prevalent in our context, and should contribute to developing and implementing food policies in universities to help reduce this public health problem. We hope that this study will be a starting point for other studies with a high level of scientific evidence, such as interventions that simultaneously address numerous sociodemographic factors to reduce the problem of FI in university students.

## Figures and Tables

**Table 1:** Frequency of food security and food insecurity according to personal sociodemographic characteristics of university students.

Personal Sociodemographic Characteristics of the Student	Total (*n* = 466)	Food Security (*n* = 247)	Food Insecurity (*n* = 219)	*p*-Value
Age, years ^1^	20 (19, 22)	20 (19, 22)	21 (19, 22)	0.348
Sex				
Woman ^2^	338 (72.5)	178 (52.7)	160 (47.3)	0.810
Man	128 (27.5)	69 (53.9)	59 (46.1)	
Marital status				
Single (unmarried, widowed, or divorced)	440 (94.4)	231 (52.5)	209 (47.5)	0.370
In a relationship (common-law or married)	26 (5.6)	16 (61.5)	10 (38.5)	
Self-ascription to an Indigenous group				
No	464 (99.6)	247 (53.2)	217 (46.8)	0.220
Yes	2 (0.4)	0 (0)	2 (100)	
School year				
1 to 2 years	296 (63.5)	163 (55.1)	133 (44.9)	0.239
3 to 5 years	170 (36.5)	84 (49.4)	86 (50.6)	
Type of student				
Undergraduate	435 (93.3)	228 (52.4)	207 (47.6)	0.339
Postgraduate	31 (6.7)	19 (61.3)	12 (38.7)	
Undergraduate area of study				
Nutrition	99 (22.8)	50 (50.5)	49 (49.5)	0.002
Medicine	89 (20.5)	57 (64)	32 (36)	
Psychology	61 (14)	21 (34.4)	40 (65.6)	
Nursing	55 (12.6)	33 (60)	22 (40)	
Physical culture and sports	51 (11.7)	25 (49)	26 (51)	
Dentistry	32 (7.4)	23 (71.9)	9 (28.1)	
Higher university technician ^3^	28 (6.4)	10 (35.7)	18 (64.3)	
Other undergraduate careers ^4^	20 (4.6)	9 (45)	11 (55)	
Type of postgraduate course				
PhD	20 (64.5)	11 (55)	9 (45)	0.452
Master’s Degree	11 (35.5)	8 (72.7)	3 (27.3)	
Receipt of scholarship				
No	395 (84.8)	210 (53.2)	185 (46.8)	0.870
Yes	71 (15.2)	37 (52.1)	34 (47.9)	
Labor activity				
No	312 (66.9)	178 (57.1)	134 (42.9)	0.013
Yes	154 (33.1)	69 (44.8)	85 (55.2)	
The student is their own financial support				
No	368 (79)	202 (54.9)	166 (45.1)	0.114
Yes	98 (21)	45 (45.9)	53 (54.1)	
A scholarship is student’s financial support				
No	441 (94.6)	230 (52.2)	211 (47.8)	0.123
Yes	25 (5.4)	17 (68)	8 (32)	
Parents/guardian are student’s financial support				
No	60 (12.9)	31 (51.7)	29 (48.3)	0.824
Yes	406 (87.1)	216 (53.2)	190 (46.8)	
Partner/relative is student’s financial support				
No	425 (91.2)	224 (52.7)	201 (47.3)	0.678
Yes	41 (8.8)	23 (56.1)	18 (43.9)	
Students’ children				
No	454 (97.4)	239 (52.6)	215 (47.4)	0.337
Yes	12 (2.6)	8 (66.7)	4 (33.3)	
Reception of food aid ^5^				
No	452 (97)	242 (53.5)	210 (46.5)	0.188
Yes	14 (3)	5 (35.7)	9 (64.3)	

^1^ the quantitative variable is expressed as median (25th, 75th percentile). Mann/Whitney U test was used to evaluate the differences between the FI categories. *p* < 0.05 was considered significant. ^2^ the qualitative variables are expressed as frequency (%). As applied, Chi-square or Fisher’s exact test was performed to evaluate the differences between the FI categories. *p* < 0.05 was considered significant. ^3^ university technician in emergencies, occupational safety and rescue, radiology and imaging, physical therapy, and respiratory therapy. ^4^ other careers include forensic science, podiatry, administration, law, physics, computer engineering, logistics and transportation engineering, pharmaceutical chemist, and biologist. ^5^ food support such as milk, food pantry, prepared foods, food supplements, vitamins, minerals, etc.

**Table 2:** Frequency of food security and food insecurity according to household sociodemographic characteristics of university students.

Household Sociodemographic Characteristics ^1^	Total (*n* = 466)	Food Security (*n* = 247)	Food Insecurity (*n* = 219)	*p*-Value
Household type				
Nuclear	277 (59.4)	155 (56)	122 (44)	0.002
Single parent	70 (15)	31 (44.3)	39 (55.7)	
Extended	57 (12.23)	33 (73.3)	12 (26.7)	
Co-resident/roommates	45 (9.7)	21 (36.8)	36 (63.2)	
Unipersonal	17 (3.7)	7 (41.2)	10 (58.8)	
Homeowner				
Own	287 (61.7)	163 (56.8)	124 (43.2)	0.127
Rented	132 (28.4)	63 (47.7)	69 (52.3)	
Borrowed	46 (9.9)	21 (45.7)	25 (54.3)	
Socioeconomic status ^2^				
A/B	164 (35.2)	97 (59.1)	67 (40.9)	0.063
C+	161 (34.6)	88 (54.7)	73 (45.3)	
C	85 (18.2)	37 (43.5)	48 (56.5)	
C−, D+, D	56 (12.0)	25 (44.6)	31 (55.4)	
Potable water at home				
Yes	459 (98.5)	245 (53.4)	214 (46.6)	0.262
No/sometimes	7 (1.5)	2 (28.6)	5 (71.4)	
Refrigerator for food storage in the house				
Yes	464 (99.6)	247 (53.2)	217 (46.8)	0.220
No/sometimes	2 (0.4)	0 (0)	2 (100)	
Stove for food preparation at home				
Yes	464 (99.6)	246 (53)	218 (47)	1.000
No/sometimes	1 (0.2)	1 (50)	1 (50)	
Gas at home				
Yes	453 (97.2)	244 (53.9)	209 (46.1)	0.045
No/sometimes	13 (2.8)	3 (23.1)	10 (76.9)	
Electricity at home				
Yes	465 (99.8)	247 (53.1)	218 (46.9)	0.470
No/sometimes	1 (0.2)	0 (0)	1 (100)	
Services and appliances always available				
Yes	450 (96.6)	244 (54.2)	206 (45.8)	0.009
No/sometimes	16 (3.4)	3 (18.8)	13 (81.2)	
Number of heads of household				
One	254 (54.5)	122 (48)	132 (52)	0.019
Two or more	212 (45.5)	125 (58)	87 (41)	
Sex of head of household				
Woman	197 (42.3)	91 (46.2)	106 (53.8)	0.012
Man	269 (57.7)	156 (58)	113 (42)	
Marital status of head of household				
Single (unmarried, widowed, or divorced)	126 (27)	61 (48.4)	65 (51.6)	0.227
In a relationship (common-law or married)	340 (73)	186 (54.7)	154 (45.3)	
Educational level of head of household				
High school/technologist or less	233 (50)	112 (48.1)	121 (51.9)	0.011
Bachelor’s degree	163 (35)	87 (53.4)	76 (46.6)	
Master’s degree/doctorate	70 (15)	48 (68.6)	22 (31.4)	
Father’s educational level				
High school/technologist or less	233 (51.1)	113 (48.5)	120 (51.5)	0.068
Bachelor’s degree	164 (36)	91 (55.5)	73 (44.5)	
Master’s degree/doctorate	59 (12.9)	38 (64.4)	21 (35.6)	
Mother’s educational level				
High school/technologist or less	263 (56.7)	133 (50.6)	130 (49.4)	0.045
Bachelor’s degree	154 (33.2)	81 (52.6)	73 (47.4)	
Master’s degree/doctorate	47 (10.1)	33 (70.2)	14 (29.8)	

^1^ the qualitative variables are expressed as frequency (%). Chi-square or Fisher’s exact Chi-square was performed to evaluate the differences between the FI categories according to applicability criteria. *p* < 0.05 was considered significant. ^2^ due to the low proportion of C−, D+, and D− levels, these were regrouped into a single category; level E had no responses.

**Table 3:** Association between university students’ sociodemographic characteristics and food insecurity.

	Food Insecurity			
Sociodemographic Characteristics	Simple Model		Multiple Model ^1^	
	OR (95% CI)	*p*-Value	OR (95% CI)	*p*-Value
Student’s work activity				
No	Ref.		Ref.	
Yes	1.64 (1.11, 2.41)	0.013	1.55 (1.03, 2.35)	0.038
The student is their own financial support				
No	Ref.	0.115	---	
Yes	1.43 (0.92, 2.24)			
A scholarship is student’s financial support				
No	Ref.		Ref.	
Yes	0.51 (0.22, 1.21)	0.129	0.46 (0.17, 1.20)	0.111
Receipt of food support ^2^				
No	Ref.		Ref.	
Yes	2.07 (0.68, 6.29)	0.197	2.27 (0.70, 7.42)	0.174
Homeowner				
Own	Ref.		Ref.	
Rented	1.44 (0.95, 2.18)	0.084	1.10 (0.65, 1.86)	0.716
Borrowed	1.56 (0.84, 2.92)	0.160	1.52 (0.77, 3.01)	0.230
Household type				
Nuclear	Ref.		Ref.	
Single parent	1.60 (0.94, 2.71)	0.082	1.00 (0.54, 1.86)	0.998
Extended	0.46 (0.23, 0.93)	0.031	0.35 (0.16, 0.76)	0.008
Co-resident/roommate	2.18 (1.21, 3.92)	0.009	1.84 (0.87, 3.90)	0.110
Unipersonal	1.81 (0.67, 4.91)	0.240	1.46 (0.49, 4.35)	0.499
Socioeconomic status				
A/B	Ref.		---	
C+	1.20 (0.77, 1.86)	0.414		
C	1.88 (1.11, 3.19)	0.020		
C−, D+, D	1.80 (0.97, 3.31)	0.061		
Number of heads of household				
One	1.55 (1.08, 2.25)	0.019	1.39 (0.90, 2.15)	0.134
Two or more	Ref.		Ref.	
Sex of head of household				
Woman	1.61 (1.11, 2.33)	0.012	1.64 (1.07, 2.51)	0.023
Man	Ref.		Ref.	
Educational level of head of household				
High school/technologist or less	2.36 (1.34, 4.15)	0.003	2.36 (1.28, 4.33)	0.008
Bachelor’s degree	1.91 (1.06, 3.44)	0.032	1.95 (1.04, 3.66)	0.038
Master’s degree/doctorate	Ref.		Ref.	
Father’s educational level				
High school/technologist or less	1.92 (1.06, 3.47)	0.030	---	
Bachelor’s degree	1.45 (0.78, 2.69)	0.235		
Master’s degree/doctorate	Ref.			
Mother’s educational level				
High school/technologist or less	2.30 (1.18, 4.50)	0.015	---	
Bachelor’s degree	2.12 (1.05, 4.28)	0.035		
Master’s degree/doctorate	Ref.			
Hosmer/Lemeshow ^3^, Chi-square	---	---	6.29	0.6144

Abbreviations: CI. confidence interval; OR. Odds Ratio; Ref. reference. ^1^ multiple logistic regression was performed from the individual sociodemographic variables that obtained a value of *p* < 0.2 in the univariate analysis with FI. In the final multiple logistic regression model, the variables educational level of the father (average VIF = 4.02) and mother (average VIF = 4.23) were eliminated because they were collinear with the academic level of the head of the household (average VIF = 4.64). Subsequently, we also eliminated the variable socioeconomic status (average VIF = 3.19) because it presented collinearity with the educational level of the head of the household (average VIF = 2.79). We decided to leave the variable head of the household in the model because it better explained the final model according to the pseudo R^2^: 7.2% [the educational level of the head of household] vs. 6.7% [socioeconomic status]. The variable regarding the student being their financial support was not included in the model because it was moderately correlated with the student’s labor activity variable (rho = 0.4882). *p* < 0.05 was considered a significant association in all the logistic regression analyses. ^2^ food support such as milk, food pantry, prepared foods, food supplements, vitamins, and minerals, etc. ^3^ the Hosmer/Lemeshow test was used to evaluate the goodness of fit between the observed and expected values. A *p* > 0.05 in this analysis indicates no disparity between these values or indicates an adequate fit.

**Table 4:** Clusters of sociodemographic characteristics.

Sociodemographic Characteristics ^1^	Total Sample (*n* = 454) ^2^	Cluster 1 (*n* = 163)	Cluster 2 (*n* = 144)	Cluster 3 (*n* = 147)	*p*-Value
Student’s work activity					
No	305 (67.2)	109 (66.9)	105 (72.9)	91 (61.9)	0.135
Yes	149 (32.8)	54 (33.1)	39 (27.1)	56 (38.1)	
Parents/guardians are student’s financial supporter					
No	58 (12.8)	33 (20.2)	16 (11.1)	9 (6.1)	0.001
Yes	396 (87.2)	130 (79.8)	128 (88.9)	138 (93.9)	
Household type					
Nuclear	276 (60.8)	18 (11)	129 (89.6)	129 (87.8)	NA ^3^
Single parent	64 (14.1)	61 (37.4)	0 (0)	3 (2)	
Co-resident/roommate	55 (12.1)	44 (27)	7 (4.9)	4 (2.7)	
Enlarged	43 (9.5)	28 (17.2)	8 (5.6)	7 (4.8)	
Unipersonal	16 (3.5)	12 (7.4)	0 (0)	4 (2.7)	
Homeowner					
Own	281 (61.9)	74 (45.4)	111 (77.1)	96 (65.3)	<0.001
Rented	128 (28.2)	71 (43.6)	23 (16)	34 (23.1)	
Borrowed	45 (9.9)	18 (11)	10 (6.9)	17 (11.6)	
Socioeconomic status ^4^					
A/B	161 (35.5)	34 (20.9)	110 (76.4)	17 (11.6)	<0.001
C+	156 (34.4)	64 (39.3)	29 (20.1)	63 (42.9)	
C	83 (18.3)	39 (23.9)	4 (2.8)	40 (27.2)	
C−, D+, D	54 (11.9)	26 (16)	1 (0.7)	27 (18.4)	
Number of heads of household					
One	243 (53.5)	136 (83.4)	49 (34)	58 (39.5)	<0.001
Two or more	211 (46.5)	27 (16.6)	95 (66)	89 (60.5)	
Sex of head of household					
Woman	188 (41.4)	124 (76.1)	30 (20.8)	34 (23.1)	<0.001
Man	266 (58.6)	39 (23.9)	114 (79.2)	113 (76.9)	
Marital status of head of household					
Single (unmarried, widowed, or divorced)	118 (26)	117 (71.8)	1 (0.7)	0 (0)	NA ^3^
In a relationship (common-law or married)	336 (74)	46 (28.2)	143 (99.3)	147 (100)	
Educational level of head of household					
High school/technologist or less	228 (50.2)	76 (46.6)	5 (3.5)	147 (100)	NA ^3^
Bachelor’s degree	159 (35)	68 (41.7)	91 (63.2)	0 (0)	
Master’s degree/doctorate	67 (14.8)	19 (11.7)	48 (33.3)	0 (0)	
Father’s educational level					
High school/technologist or less	233 (51.3)	80 (49.1)	14 (9.7)	139 (94.6)	<0.001
Bachelor’s degree	163 (35.9)	66 (40.5)	89 (61.8)	8 (5.4)	
Master’s degree/doctorate	58 (12.8)	17 (10.4)	41 (28.5)	0 (0)	
Mother’s educational level					
High school/technologist or less	259 (57)	80 (49.1)	44 (30.6)	135 (91.8)	<0.001
Bachelor’s degree	150 (33)	73 (44.8)	66 (45.8)	11 (7.5)	
Master’s degree/doctorate	45 (9.9)	10 (6.1)	34 (23.6)	1 (0.7)	

^1^ all the qualitative variables are expressed as frequency (%). A Chi-square was performed to evaluate the differences between the categories. *p* < 0.05 was considered significant. ^2^ the cluster analysis only considered the participants who have data in all the variables of interest. ^3^ more than 20% had an expected frequency of less than 5. ^4^ due to low proportions, levels C−, D+, and D− were regrouped into a single category; level E had no responses.

**Table 5:** Association between the cluster of university students’ sociodemographic characteristics and food insecurity.

Clusters of Sociodemographic Characteristics	Food Insecurity	
	OR (95%CI) ^1^	*p*-Value
Cluster 1	1.96 (1.24, 3.09)	0.004
Cluster 2	Ref.	
Cluster 3	1.52 (0.95, 2.42)	0.082

Abbreviations: CI. confidence interval; OR. Odds Ratio; Ref. reference. ^1^ the association between the clusters of sociodemographic characteristics (independent variable) and FI (dependent variable) was analyzed with the logistic regression analysis. *p* < 0.05 was considered significant.

## Data Availability

The data presented in this study is available upon request from the corresponding author due to privacy.

## Supplementary Materials

The following supporting information can be downloaded at: https://www.mdpi.com/article/10.3390/foods13162507/s1, Figure S1: Importance of sociodemographic characteristics in the Two-Step cluster analysis; Table S1: Simple logistic regression model of each sociodemographic characteristic of university students associated with food insecurity.
