# Supplementary material for: Clusters of Sociodemographic Characteristics and Their Association with Food Insecurity in Mexican University Students
Source: Foods. 2024 Aug 10;13(16):2507. doi: 10.3390/foods13162507 (PMC11353896; doi:10.3390/foods13162507)
Supplement: Supplementary file 1 [file foods-13-02507-s001.zip › foods-3100015-supplementary.pdf]

Table S1. Simple logistic regression model of each sociodemographic characteristic of university students associated with food insecurity.

Sociodemographic characteristics	Simple model[1]	
	OR (95% CI)	p
Age, years	1.01 (0.95, 1.06)	0.785
Sex		
Woman	1.05 (0.70, 1.58)	0.810
Man	Ref.	
Marital status		
Single (unmarried, widowed or divorced)	1.45 (0.64, 3.26)	0.372
In a relationship (common-law or married)	Ref.	
School year		
Year 1 to 2	Ref.	
Year 3 to 5	1.25 (0.86, 1.83)	0.239
Type of student		
Undergraduate	1.44 (0.68, 3.03)	0.341
Postgraduate	Ref.	
Receipt of scholarship		
No	0.96 (0.58, 1.59)	0.870
Yes	Ref.	
Labor activity		
No	Ref.	
Yes	1.64 (1.11, 2.41)	0.013
The student is his own financial support		
No	Ref.	
Yes	1.43 (0.92, 2.24)	0.115
A scholarship is student's financial support		
No	Ref.	
Yes	0.51 (0.22, 1.21)	0.129
Parents/guardian are student's financial support		
No	Ref.	
Yes	0.94 (0.55, 1.62)	0.824
Partner/relative is student's financial support		
No	Ref.	
Yes	0.87 (0.46, 1.66)	0.678
Sons		
No	Ref.	
Yes	0.56 (0.17, 1.87)	0.343
Reception of food aid[2]		
No	Ref.	
Yes	2.07 (0.68, 6.29)	0.197
Household type		
Nuclear	Ref.	
Single parent	1.60 (0.94, 2.71)	0.082
Enlarged	0.46 (0.23, 0.93)	0.031

Co-resident/roommates	2.18 (1.21, 3.92)	0.009
Unipersonal	1.81 (0.67, 4.91)	0.240
Housing tenure		
Own	Ref.	
Rented	1.44 (0.95, 2.18)	0.084
Borrowed	1.56 (0.84, 2.92)	0.160
Socioeconomic status [3]		
A/B	Ref.	
C+	1.20 (0.77, 1.86)	0.414
C	1.88 (1.11, 3.19)	0.020
C-, D+, D	1.80 (0.97, 3.31)	0.061
Number of heads of household		
1	1.55 (1.08, 2.25)	0.019
2 or more	Ref.	
Gender of head of household		
Woman	1.61 (1.11, 2.33)	0.012
Man	Ref.	
Marital status of head of household		
Single (unmarried, widowed or divorced)	1.29 (0.85, 1.94)	0.227
In a relationship (common-law or married)	Ref.	
Educational level of head of household		
High school/technologist or less	2.36 (1.34, 4.15)	0.003
Bachelor's Degree	1.91 (1.06, 3.44)	0.032
Master's Degree/Doctorate	Ref.	
Father's educational level		
High school/technologist or less	1.92 (1.06, 3.47)	0.030
Bachelor's Degree	1.45 (0.78, 2.69)	0.235
Master's Degree/Doctorate	Ref.	
Mother's educational level		
High school/technologist or less	2.30 (1.18, 4.50)	0.015
Bachelor's Degree	2.12 (1.05, 4.28)	0.035
Master's Degree/Doctorate	Ref.	

Abbreviations: CI. Confidence interval; OR. Odds Ratio; Ref. Reference.

[1] Simple logistic regression was performed to assess association between sociodemographic variables and food insecurity. $p<0.05$ was considered a significant association.

[2] Food support such as milk, food pantry, prepared foods, food supplements, vitamins and minerals, financial support, etc.

[3] Due to low proportions, levels C-, D+ and D- were regrouped into a single category; there were no responses from level E.

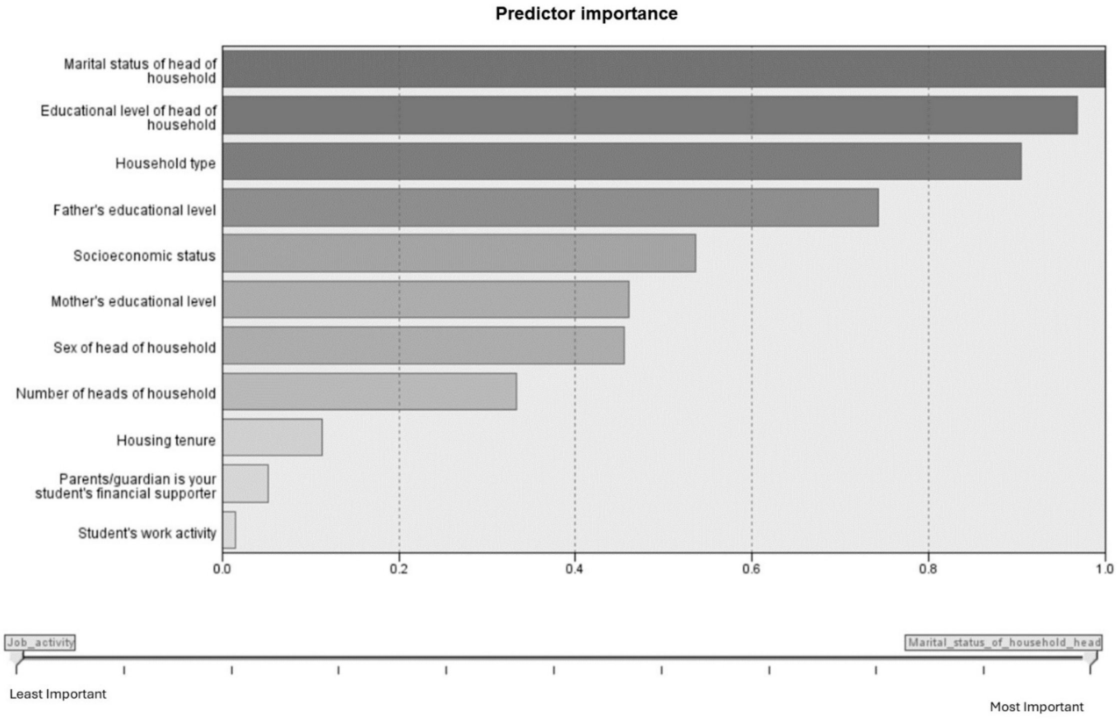

Figure S1. Importance of sociodemographic characteristics in the Two Steps Cluster analysis.

The bar chart shows the sociodemographic variables that formed the clusters, in order of importance (from top to bottom).
